# Contamination of microbubbles of air may occur at all investigated measurement points during hemodialysis

**DOI:** 10.1177/03913988251334953

**Published:** 2025-05-04

**Authors:** Per Jonsson, Bernd Georg Stegmayr

**Affiliations:** Department Public Health and Clinical Medicine, Unit Medicine, Umea University, Umea, Sweden

**Keywords:** Hemodialysis, hemodiafiltration, microbubbles, air contamination, adverse events

## Abstract

Microbubbles (MBs) of air occur in the hemodialysis (HD) extracorporeal circuit and may enter the bloodlines of the patient. The aim of the present study was to investigate possible sites of contamination. Seventeen patients performed 20 HD (Baxter AK200S *n* = 5 and Artis *n* = 15) and 930 ultrasound measurements of MBs/min (Hatteland CMD10 device). Detection ranges were diameters between 2.5 and 50 µm. Hemodiafiltration with postdilution (HDF-post) was performed in 14 dialyses, predilution (HDF-pre) in 1 dialysis, and HD using hemocontrol (HDhc) in 5 dialyses. Measurement points were M1—after the blood access, M2—before the dialyzer, M3—after the dialyzer, and M4—after the venous chamber. At each point, 10 measures of MBs were performed. MB contamination of the blood was larger at all points when the access was an arteriovenous fistula compared to a central dialysis catheter (*p* < 0.001). MB levels with the AK200 versus the Artis were lower at M1, higher at M2 (*p* ⩽ 0.005), and were similar at M3 and M4. HDF-pre had fewer MBs than HDF-post, whereas HDhc had more MBs than HDF-post (*p* < 0.001). An increase of MBs was seen at M2 during an internal “Autotest.” No air alarms were induced during dialyses. MBs were detected in the extra corporeal circuit at all points investigated. The venous chambers used did not significantly reduce contamination. The detected MBs did not induce air alarms when the blood returned to the patient.

## Introduction

The life span of patients in hemodialysis (HD) are limited due to the retention of uremic toxins and fluid between dialyses as well as to cardiac stress^1,2^ and air contamination through the extracorporeal circuit (ECC). These microbubbles (MBs) of air pass the venous chambers into the bloodline of the patient^3–10^; here a number of them deposit as air emboli as well as induce microemboli in the lungs, heart, and brain.^11–13^ Tissue damage induced by the emboli is accumulated in the body during each HD.

Problems with air contamination were noted early on after death and were attributed to air embolies during hemodialysis.^14^ This resulted in the development of venous chambers and air traps.^15^ MBs that passed the air trap were assumed to be adsorbed. Further investigations were initiated after notice of air contamination in the ECC that appeared as visual foam that entered through the bloodline into the patient.^16^ In vitro studies using a viscous and transparent fluid verified that visible MBs of air could pass the air trap without inducing an alarm or a pump stop of the safety system that housed the venous chamber and air detector system. Several in vitro and clinical studies using ultrasound detectors confirmed MBs in the return line to the patient.^3–10^ These MBs were mostly small sizes^9^ but even those above 500 µm diameter were verified.^10^ Insufficient automatic priming of the ECC caused foam in the venous chambers that was mainly derived from the dialyzer and especially after changing from the dialysis mode to hemodiafiltration.^17^ The detected MBs were reduced after upgrade of the priming program.^17^ X-ray verified that the dialyzer was an important location where air could be hidden. Another cause of air contamination was insufflation of air into the ECC that appeared through an untight access connection.^6^ This was revealed after data analysis that showed a straight high leveled line of air contamination.^6^ No safety alarm was activated.

A Swedish risk database contains cases of suspected air pollution in the return blood line that affected several patients who had fallen ill and had symptoms. Air alarms were activated in some cases but not in others. The manufacturer judged the situation safe, but could not explain the observation or the air alarm without visible indication of air.^18^

Several reports on air emboli have been published.^14,19–27^

Air present in the blood of the extracorporeal dialysis circuit is regarded as a material that affects the blood as it circulates through the dialysis unit and promotes coagulation. Air infusion or infusion of microbubbles of air is another type of risk where the material air in ISO 10993-18^28^ can be seen as a leachable material that is released from the device and infused into the patient’s vessels. In the vessels, it continues to trigger coagulation.^13^ The air MB contamination into the vessels is present during every extracorporeal treatment. Chronic HD constitutes repeated risk exposure, that is, three times/week, in many patients for the remaining life. Various modes of dialysis exist such as hemodialysis (HD), predilution hemodiafiltration (HDF), postdilution HDF, and mixed HDF.^29^ In a recent study, the exposure to air bubbles differed between HD and HDF for the Fresenius 5008 bloodline and for a new Emboless^®^ bloodline.^30^

Since previous studies have not systematically investigated the origin of MBs that enter the blood within the ECC, it is deemed necessary to perform a more in-depth investigation in clinical practice to clarify variables that contribute to the contamination.

The primary aim of the present prospective clinical study was to systematically investigate possible sites of MB contamination. The secondary aim was to study the possible air contamination sites in the frequently used Artis and AK200 systems for patients undergoing HD and HDF.

## Materials and methods

Seventeen patients (mean age = 68 years) performed 20 HDs with various dialysis settings. Patients were informed and consented to participate in the study. The study was approved by the Umea Ethical Committee (Dnr 05-138M).

The following two types of dialysis systems were used: the Gambro AK200S together with the Gambro Blood Tubing System, BL 200BD PREPOST, 114,536 (*n* = 5 dialyses); and the Gambro Artis together with the bloodline ArtiSet, ARTISET PREPOST, 11,5281 (*n* = 15 dialyses). The Artis bloodline contains a closed system for arterial and venous pressure measurements using pressure pods; the AK200S maintains open air-filled tube connections for distributing the pressure to the pressure transducer.

The AK 200 Ultra S systems were manually primed using 1.6 L online prepared fluid. The Artis systems were primed with an automatic mode using fluid online produced by the Artis machine 3.0 L (13 dialyses) or 4.0 L (5 dialyses). MB measurements were taken 930 times during high-efficiency dialysis and hemodiafiltration treatments.

**Figure 1.**
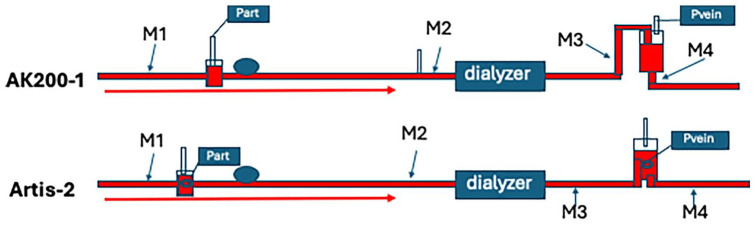
A systematic bloodline is shown for the AK200-1 and the Artis-2 devices with measurement points marked as M1 (after arterial access), M2 (before the dialyzer), M3 (after the dialyzer), and M4 (after the venous chamber/air trap). At each point, 10 measurements of 1 minute each were made. Connection sites for pressure measurement of the arterial (P_art_) and venous sites (P_vein_).

Recording of MBs were done with a Hatteland CMD10 bubble detector device that uses an ultrasound doppler and a special probe for dialysis bloodlines that measured air MBs between 2.5 and 50 µm. Blood flow (blood pump speed ml/min) in the dialysis system was kept constant during dialysis when measurements were made at four points. The four measurement points were at the following locations: after exit of the artery needle or CDC—after the blood access (M1), before the dialyzer (M2), after the dialyzer (M3), and after the venous chamber (M4; Figure 1). At each point, 10 subsequent measurements were taken, each for 1 min (10 min × 1 min). Results were registered as MBs/min. Treatment modes were hemodiafiltration with postdilution (HDF-post) in 14 dialyses, and predilution (HDF-pre) in 1 dialysis. Five HDs were performed with the Artis and the dialyzer PF140H using hemocontrol (HDhc).

**Figure 2A.**
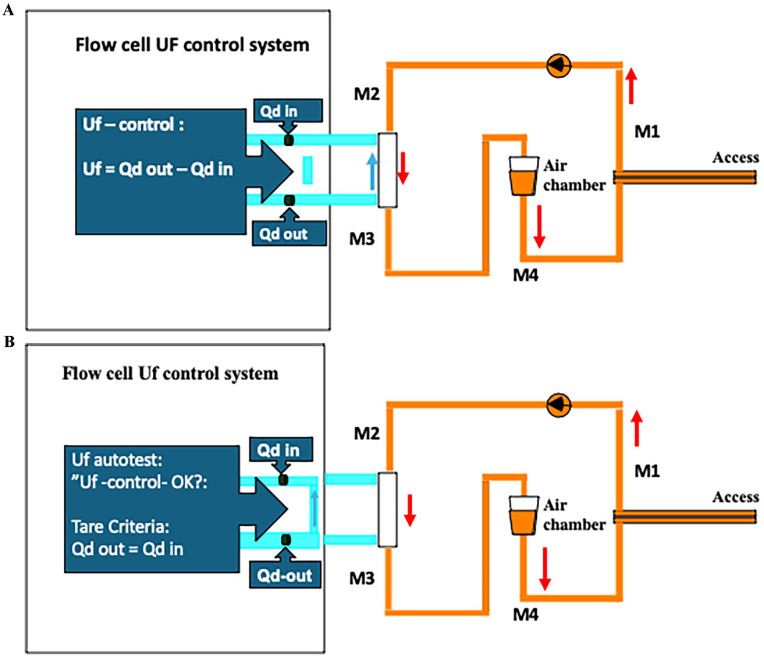
Ultrafiltration (Uf) system: The fluid balance system in the Baxter Artis and AK200 uses a control system that measures the volume of dialysate into the dialyzer (Qd-in) and the volume out from the dialyzer (Qd-out). The control system regulates the Qd-in and Qd-out during the treatment to reach the target ultrafiltration (Uf) volume. **Figure 2B**. To minimize ultrafiltration regulation error, the measuring channels Qd-in and Qd-out during “Autotest” are tared automatically at intervals and according to internal criteria in the device. During these tarings, flow and pressure conditions in the dialyzer change. Data indicate that the microbubbles in the blood tubes increase during taring (see Supplemental Figure 1).

Events such as automatic tests performed by the dialysis device were noted (Figure 2A and 2B).

Calculations were performed using means (±1SD), medians, and interquartile ranges (IQR). Mann-Whitney *U* test was used for comparison between groups and Wilcoxon used when comparing pairs. Spearman’s test (*rho*) was used for bivariate correlation analyses. Multiple stepwise regression analysis was performed to investigate possible risk factors for microbubble counts at the different measurement points (M1–M4). Variables included in the model were needle size (Gauge), blood pump speed (ml/min), artery pressure (mmHg, pressures measured before the blood pump), venous pressure (mmHg, at the site of venous chamber), transmembrane pressure (TMP, mmHg), type of device, dialysate temperature, type of treatment (HDF-post, HDF-pre, and HDhc), priming volume (ml), and hour of dialysis when the measurement was made (i.e. 1–6 h). A two-tailed *p*-value <0.05 was considered as significant. The IBM software SPSS version 28.0 was used.

## Results

Baseline conditions of variables during measurement of MBs are shown in Supplemental Table 1 and Supplemental Tables 2A–2D. Table 1 shows the overview of MBs/min at the different measurement points.

**Table 1.** Results of microbubbles/min at different measurement points by the Artis and AK200 dialysis devices, and during various modes of treatment.

Device and mode	*N*	*M*	SD	Median	Percentiles	
					25	75
Artis—Hemocontrol						
After the access	60	39.2	89.6	3	1	7
Before dialyzer	60	10.5	16.2	3	1	11
After dialyzer	60	1.52	1.83	1	0	2.75
After venous chamber	60	1.30	1.53	1	0	2
Artis—HDF predilution						
After the access	10	5.7	1.83	5.5	4.5	7.25
Before dialyzer	10	3.3	1.83	3	2.75	3.25
After dialyzer	10	0.7	0.67	1	0	1
After venous chamber	10	0.9	0.99	1	0	1.25
Artis—HDF postdilution						
After the access	110	2.5	3.30	1.5	0	3
Before dialyzer	110	5.61	6.46	3	0	9.25
After dialyzer	110	1.37	2.88	0	0	1.25
After venous chamber	110	0.67	1.33	0	0	1
AK200—HDF postdilution						
After the access	50	1.9	2.46	1	0	3.25
Before dialyzer	60	16.0	23.6	6.5	2.25	12
After dialyzer	50	0.76	1.08	0	0	1
After venous chamber	50	1.7	3.27	0	0	2

Measurements were done before the blood pump right after the access (M1), before the dialyzer (M2), after the dialyzer (M3), and after the venous chamber (M4).

Table 2 shows the statistical comparisons of MB contamination of the bloodline at different locations and for different conditions. MB contamination was larger for patients using AVF at all points (M1–M4) compared to those with a central dialysis catheter (CDC; *p* ⩽ 0.005). A higher number of MBs was present at M1 for those who had an AVF and an access needle of a small diameter. The mean artery pressure was in the same range for AVF and CDC (−185 ± 42 vs −209 ± 19, *p* = 0.24) despite a lower blood pump speed with CDC (452 ± 72 vs 364 ± 31, *p* < 0.001).

**Table 2.** Mann-Whitney *U* test comparison of counts of microbubbles/min at all sites combined, or at separate sites: after the access (M1), before the dialyzer (M2), after the dialyzer (M3), and after the venous chamber (M4).

Data origin	Variable	Comparators	Mann-Whitney *U* test *p*-value
Access	All data sites	AVF vs CDC	0.000
Needle sizes	All data sites	15 vs 17 Gauge	0.059
Needle sizes	All data sites	17 vs 14 Gauge	0.001
Needle sizes	All data sites	15 vs 14 Gauge	0.027
Priming volume	All data sites	1.6 vs 3.0	0.200
Priming volume	All data sites	1.6 vs 4.0	0.428
Priming volume	All data sites	3.0 vs 4.0	0.020
Device	All data sites	AK200 vs Artis	0.495
All data	Sites	M2 vs M1	<0.001
All data	Sites	M2 vs M3	<0.001
All data	Sites	M3 vs M4	0.294
Access	AVF vs CDC	M1	0.000
Access	AVF vs CDC	M2	0.000
Access	AVF vs CDC	M3	0.000
Access	AVF vs CDC	M4	0.005
Needle size	15 vs 14 Gauge	M1	0.053
Needle size	15 vs 17 Gauge	M1	0.000
Needle size	17 vs 14 Gauge	M1	0.000
Needle size	15 vs 14 Gauge	M2	0.000
Needle size	15 vs 17 Gauge	M2	0.098
Needle size	17 vs 14 Gauge	M2	0.000
Needle size	15 vs 14 Gauge	M3	0.198
Needle size	15 vs 17 Gauge	M3	0.819
Needle size	17 vs 14 Gauge	M3	0.304
Needle size	15 vs 14 Gauge	M4	0.025
Needle size	15 vs 17 Gauge	M4	0.141
Needle size	17 vs 14 Gauge	M4	0.628
Devices	Artis vs AK200	M1	0.005
Devices	AK200 vs Artis	M2	0.001
Devices	Artis vs AK200	M3	0.321
Devices	Artis vs AK200	M4	0.545
Dialysis mode	HDhc. vs HDF-post	All data sites	0.001
Dialysis mode	HDhc. vs HDF-pre	All data sites	0.651
Dialysis mode	HDF-post vs HDF-pre	All data sites	0.028
Dialysis mode	HDhc. vs HDF-post	M1	0.000
Dialysis mode	HDhc. vs HDF-post	M2	0.564
Dialysis mode	HDhc. vs HDF-post	M3	0.024
Dialysis mode	HDhc. vs HDF-post	M4	0.007
Dialysis mode	HDF-pre vs HDF-post	M1	0.000
Dialysis mode	HDF-post vs HDF-pre	M2	0.502
Dialysis mode	HDF-post vs HDF-pre.	M3	0.691
Dialysis mode	HDF-post vs HDF-pre	M4	0.240
Devices	AK200	M3 vs M4	0.669
Devices	Artis	M3 vs M4	0.148

AVF: arterio-venous fistula; CDC: central dialysis catheter; HDhc.: Hemocontrol; HDF-pre: HDF predilution; HDF-post: HDF postdilution.

The variable with the highest MB contamination is given first.

Overall MB contamination was similar between those treated with the AK200 versus the Artis, but the Artis had a higher number of MBs at M1 and the AK200 a higher number of MBs at M2.

MB contaminations at M1, M3, and M4 were higher with the dialysis mode using HDhc versus HDF-post. Those treated with HDF-pre mode had more MBs than those with HDF-post. There was no difference in MBs between measurement points M3 and M4 for either of the devices.

There was no difference in MBs at M3 (after the dialyzer) versus at M4 (after the venous chamber).

Analyses of non-parametric bivariate correlation between variables are shown in Supplemental Tables 3A and 3B.

Multiple stepwise regression analyses with the MBs at the different measurement points as the dependent variable are shown in Supplemental Tables 4A–4E. The MBs/min at M1 showed a significant model (*r* = 0.522, *p* = 0.023) that included the variables type of dialysis mode, blood pump speed, artery pressure, venous pressure, and size of needle diameter. The MBs/min at M2 showed a significant model (*r* = 0.486, *p* < 0.001) that included the venous pressure. The MBs/min at M3 (after the dialyzer) showed a significant model (*r* = 0.673, *p* = 0.037) that included the artery pressure, venous pressure, TMP, dialysate temperature, and type of device. The MBs/min at M4 (after the venous chamber) showed a significant model (*r* = 0.485, *p* < 0.001) that included venous pressure, artery pressure, and type of device.

## Specific episodes

### Reduction in Qb

In one patient performing HDF-post, a follow-up measurement was performed directly in conjunction with the first series after having reduced the blood pump speed from 390 ml/min to 330 ml/min (using AK200), while no other change was made. Then the artery pressure changed from −127 to −90 mmHg (*p* = 0.002), the venous pressure changed from 199 to 162 mmHg (*p* = 0.002), the TMP changed from 199 to 248 mmHg (*p* = 0.002), and the MB counts at the different sites changed as follow: at M1 (*n* = 10, 0.7–0/min, *p* = 0.008), at M2 (5.4–1.6, *p* = 0.052), at M3 (0.4–0.0, *p* = 0.046), and at M4 (0.4–0.1, *p* = 0.18).

### Autotest of the device ultrafiltration measuring system

In one patient performing HDF-post with the AK200, a follow-up measurement was performed at the measurement point M2 directly after finalizing the first series. During the first series, the device performed an Autotest during the M2 measurement. The MBs/min was 65 (±16.3) versus 12 (±7.6; *p* = 0.005) at follow-up (Supplemental Figures 1A–C).

### Untight Luer connection between the arterial needle and the arterial bloodline was detected after measurement

A high number of MBs/min were detected at M1 but were less pronounced at M2, whereas at M3 and M4 the numbers were similarly low (Supplemental Figure 2).

No air contamination alarm was induced during any of the study dialyses.

## Discussion

The present study verified MBs at all measured sites. After the access at the M1 site, those with an AVF showed more MB counts at all measurement points than those with a CDC. The difference can be attributed to the wide diameter at the connection to the ECC with the CDC, compared to the AVF, which may cause a less influenced negative artery pressure. Here it seems that the CDC Luer locks were better connected than for the AVF. However, more negative artery pressure existed, indicating that narrowed artery lumen by thrombosis were frequent. This is similar to using the needle with the smallest diameter that showed more MB counts especially at measurement points M1 and M2. Insufflation of air through the Luer connection at the access site is facilitated by a more negative arterial pressure built up between the needle and the blood pump, especially when the blood pump speed is increased (Supplemental Tables 3A and 3B). This also indicates that the connection between the needle and the ECC bloodline needs to be sufficiently tight (Supplemental Figure 2) in relation to the negative pressure. Even using CDC as access, MBs were detected at site M1, again with a greater risk when the negative pressure was higher. However, air can also be insufflated from the artery pressure line and the heparin infusion line, if present in the bloodline but not in use, such as when low molecular weight heparin (LMWH) is administered separately. The AK200 has an open system (air filled tube) while the Artis has a sealed system (pressure pod) without blood-air contact for artery and venous pressure measurements. An open system may allow air to be sucked into the bloodline and may explain the increase of MBs at site M2 versus site M1. The MBs before the dialyzer were overall reduced at site M3 after having entered the dialyzer. Either they were stuck as emboli or merged into larger bubbles, or both. In a previous study, we noted that the shift from HD to HDF could release MBs from the dialyzer into site M3. In that study, the dialyzer contained and released extensive numbers of MBs that even built up into foam in the venous chamber.^17^ Despite priming, the dialyzers used had retained residual air.

The MBs at site M3 did not change in number at site M4, that is, after passing the venous chamber of either of the two types of venous chambers (air traps). This points to a low MB elimination capacity of the venous chambers in use. Such limited capacity was also found for these systems in an in vitro study^31^ that showed that small air bubbles that entered these types of venous chambers had a restricted chance to be eliminated from the bloodline and instead entered the return bloodline of the patient.

In the present study during the Autotest, an increase in MB counts may appear. The responsible manufacturers could (or should) check the reasons for MB contamination that appears during the machine induced Autotest.

### Limitations of the study

The MBs measured with the used devices had small diameters. A consequence is a small lift force to escape from the bloodlines with the shape of the venous chambers used, such as mentioned by Jonsson et al.^31^ We know from another study that numerous MBs above the measuring range (2.5–50 µm) will not be measured in the present setting, since many larger MBs exist, even above 500 µm. In that study, no safety alarm was initiated by the venous chambers.^10^ In addition, the present clinic uses high blood pump speed in their dialysis prescriptions, which is a negative effect for MB elimination as shown by us previously.^3,6,31,32^ The time limit of a measuring sequence of 10 min may appear short in relation to a whole dialysis session, and may allow variations of MBs. However, it was possible to analyze many more dialyses conditions during the same dialysis session. This enabled the present study to measure MB contamination at all measurement points.

The clinical value of this study is the knowledge of numerous sites where air can enter the extracorporeal system, and in addition the fact that the MBs may pass beyond the venous chamber into the return bloodline of the patient. It is known from other studies that such air is not fully absorbed in the body and may deposit as microemboli in organs such as the lungs, heart, and brain and cause repeated damage with each dialysis.^13^ Since the present alarm system of dialysis devices is not induced even upon quite high amounts of MB, we recommend regular controls of the CDC for damage. Accesses connections to the bloodline need to be well-tightened; however, not as much as to crack them. If single doses of LMWH administration are used, a bloodline without a heparin line or a fluid filled heparin line is recommended. Air blocked pressure monitors for artery and venous pressure will further restrict contamination. In addition, a lower blood pump speed (less reduced negative pressure) and well primed dialyzers will reduce exposure. However, the crucial part of the bloodline is the venous chamber and the alarming system that needs to be extensively improved. In the case of leakage of air bubbles into the dialyzed blood, by known or unknown factors, efficient air reduction in the venous chamber is the crucial barrier before blood returns to the patient. An efficient reduction also enables the use of a more sensitive air alarm system. The bubble reduction efficiency in venous chambers is limited.^31^ However, the state of the art in reduction of air microbubbles makes it possible to improve.^30^

In conclusion, microbubbles contaminate the bloodline at all points measured. The Artis and AK200 had similar MB contaminations. The venous chambers investigated in the present study do not reduce the contamination significantly. In none of the procedures was an air alarm triggered.

## Supplemental Material

sj-pdf-1-jao-10.1177_03913988251334953 – Supplemental material for Contamination of microbubbles of air may occur at all investigated measurement points during hemodialysis Supplemental material, sj-pdf-1-jao-10.1177_03913988251334953 for Contamination of microbubbles of air may occur at all investigated measurement points during hemodialysis by Per Jonsson and Bernd Georg Stegmayr in The International Journal of Artificial Organs
