# Supplementary material for: Contamination of microbubbles of air may occur at all investigated measurement points during hemodialysis
Source: Int J Artif Organs. 2025 May 4;48(5):310–7. doi: 10.1177/03913988251334953 (PMC12152285; doi:10.1177/03913988251334953)
Supplement: sj-pdf-1-jao-10.1177_03913988251334953 – Supplemental material for Contamination of microbubbles of air may occur at all investigated measurement points during hemodialysis [file sj-pdf-1-jao-10.1177_03913988251334953.pdf]

Supplemental material

Supplement Table 1: Baseline data of dialysis conditions that were present during the 930 measurements.

Variables	N	Mean	SD	Median	Minimum	Maximum	Percentile	
							25	75
Dialysate temperature, C^{o}	930	36.1	0.2	36	36	36.8	36	36
Blood pump speed, mL/min	930	436	73	400	320	550	375	520
Priming volume, mL	930	2.899	805	3.000	1.600	4.000	3.000	3.000
TMP, mmHg	930	138	101	176	-34	265	20	222
HD time of study, hour	930	2.6	0.8	3	1	4	2	3
Artery pressure, mmHg	930	-192	38	-205	-237	-90	-219	-178
Venous pressure, mmHg	930	188	21	192	149	225	168	207
Needle size, Gauge	730	14.7	0.7	15	14	17	14	15

TMP: trans membrane pressure

Supplement Table 2A-E
Distribution of various conditions during the measurements of MBs. A) Dialysate temperature, B) Priming volume, C) Hour of HD when the measurement was done, D) Access used by the various patients during the measurements, and E) Needle sizes in use during dialyses when data collection was done.

2A: Dialysate temperature

	Degree Celsius	Frequency	Percent	Valid Percent	Cumulative Percent
Valid	36.0	810	87.1	87.1	87.1
	36.3	40	4.3	4.3	91.4
	36.5	40	4.3	4.3	95.7
	36.8	40	4.3	4.3	100
	Total	930	100	100	

2B: PrimingVolume

	mLiters	Frequency	Percent	Valid Percent	Cumulative Percent
Valid	1.600	210	22.6	22.6	22.6
	3.000	520	55.9	55.9	78.5
	4.000	200	21.5	21.5	100
	Total	930	100	100	

2C: Hours on dialysis (1-6)

	Hours	Frequency	Percent	Valid Percent	Cumulative Percent
Valid	1	80	8.6	8.6	8.6
	2	330	35.5	35.5	44.1
	3	400	43	43	87.1
	4	120	12.9	12.9	100
	Total	930	100	100	

2D: Access Central dialysis catheter (CDC), Arterio-venous fistula (AVF)

	Type	Frequency	Percent	Valid Percent	Cumulative Percent
Valid	CDC	200	21.5	21.5	21.5
	AVF	730	78.5	78.5	100
	Total	930	100	100	

2E: Needle size

	Gauge	Frequency	Percent	Valid Percent	Cumulative Percent
Valid	14	320	34.4	43.8	43.8
	15	370	39.8	50.7	94.5
	17	40	4.3	5.5	100
	Total	730	78.5	100	
Missing	System	200	21.5		
Total		930	100		

Supplement Table 3A

Spearman's correlation analyses between microbubbles/min and variable data at all four sites of the bloodline combined, as well as at the different sites in relation to different variables such as blood pump speed (Qb), trans membrane pressure (TMP), priming volume, dialysis time (range 1-6h).

Measurements were performed after the access (M1), before the dialyzer (M2), after the dialyzer (M3), after the venous chamber (M4).

The number (N) of measurements, *rho* and two-tailed p-values (p) are given.

Variables		All sites	M1	M2	M3	M4
Qb	*rho*	0.247**	0.341**	0.216**	0.380**	0.139*
	p	<0.001	<0.001	<0.001	<0.001	0.035
	N	930	230	240	230	230
TMP	*rho*	-0.121**	-0.290**	-0.067	-0.086	-0.157*
	p	<0.001	<0.001	0.303	0.193	0.017
	N	930	230	240	230	230
Priming volume	*rho*	0.026	0.220**	-0.166*	0.149*	0.028
	p	0.42	<0.001	0.01	0.024	0.673
	N	930	230	240	230	230
Hour of dialysis	*rho*	-0.129**	0.009	-0.210**	-0.154*	-0.225**
	p	<0.001	0.894	0.001	0.019	<0.001
	N	930	230	240	230	230
Artery pressure	*rho*	-0.075*	-0.148*	0.075	-0.214**	-0.137*
	p	0.023	0.025	0.247	0.001	0.037
	N	930	230	240	230	230
Venous pressure	*rho*	0.087**	0.354**	-0.026	0.053	-0.1
	p	0.008	<0.001	0.685	0.422	0.131
	N	930	230	240	230	230
Dialysate temp.	*rho*	0.047	0.180**	0.138*	0.057	-0.095
	p	0.151	0.006	0.033	0.392	0.149
	N	930	230	240	230	230
Device AK200 vs Artis	*rho*	-0.022	0.186**	-0.220**	0.066	-0.04
	p	0.495	0.005	<0.001	0.322	0.546
	N	930	230	240	230	230
Needle size, Gauge	*rho*	0.117**	0.041	0.331**	0.108	0.115
	p	0.002	0.587	<0.001	0.147	0.123
	N	730	180	190	180	180

Device AK200 is given the value 1, Artis value 2;

Supplement Table 3B

Correlation analyses between microbubbles (MBs) measured separately for Artis and AK200, respectively. The N of measurements, *rho* and two-tailed p-values (p) are given.

		Artis device					AK200 device			
Variables		M1	M2	M3	M4		M1	M2	M3	M4
Qb	*rho*	0.182*	0.393**	0.332**	0.208**		0.594**	0.173	0.619**	0.06
	P-value	0.014	<0.001	<0.001	0.005		<0.001	0.186	<0.001	0.678
	N	180	180	180	180		50	60	50	50
TMP	*rho*	-0.292**	-0.03	-0.077	-0.14		-0.125	-0.603**	-0.106	-0.475**
	P-value	<0.001	0.69	0.303	0.061		0.386	<0.001	0.463	<0.001
	N	180	180	180	180		50	60	50	50
Priming volume	*rho*	0.139	0.027	0.175*	0.114		.	.	.	.
	P-value	0.064	0.718	0.019	0.128		.	.	.	.
	N	180	180	180	180		50	60	50	50
Dialysis time	*rho*	-0.219**	-0.171*	-0.336**	-0.269**		0.528**	0.051	0.594**	0
	P-value	0.003	0.022	<0.001	<0.001		<0.001	0.699	<0.001	1
	N	180	180	180	180		50	60	50	50
Artery pressure	*rho*	0.052	-0.037	-0.204**	-0.217**		-0.661**	-0.280*	-0.445**	-0.169
	P-value	0.484	0.622	0.006	0.003		<0.001	0.03	0.001	0.24
	N	180	180	180	180		50	60	50	50
Venous pressure	*rho*	0.254**	0.093	-0.068	-0.051		0.508**	-0.208	0.256	-0.217
	P-value	<0.001	0.217	0.363	0.495		<0.001	0.111	0.073	0.13
	N	180	180	180	180		50	60	50	50
Dialysate temp.	*rho*	0.167*	0.215**	0.049	-0.1		.	.	.	.
	P-value	0.025	0.004	0.517	0.181		.	.	.	.
	N	180	180	180	180		50	60	50	50
Needle size, Gauge	*rho*	0.268**	0.374**	0.236**	0.116		-0.682**	0.092	-0.475**	0.115
	P-value	0.001	<0.001	0.005	0.173		<0.001	0.525	0.002	0.478
	N	140	140	140	140		40	50	40	40
Access, CDC vs AVF	*rho*	-0.532**	-0.592**	-0.362**	-0.216**		-0.178	-0.127	-0.424**	-0.08
	P-value	<0.001	<0.001	<0.001	0.004		0.217	0.334	0.002	0.578
	N	180	180	180	180		50	60	50	50

Access CDC and AVF were given the values 1 and 2, respectively.

Supplement Table 4 A, B, C, D: Multiple stepwise regression analysis of variables with possible relation to microbubble count at different sites of measurement (M1 to M4)

Table 4A: Model Summary[a]: Measure point = M2 – before the dialyzer									
Model	R	R Square	Adjusted R Square	Std. Error of the Estimate	Change Statistics				
					R Square Change	F Change	df1	df2	Sig. F Change
1	0.360b	0.13	0.125	50.733	0.13	26.481	1	178	<0.001
2	0.444c	0.197	0.188	48.854	0.068	14.955	1	177	<0.001
3	0.476d	0.226	0.213	48.097	0.029	6.616	1	176	0.011
4	0.501e	0.251	0.234	47.463	0.025	5.729	1	175	0.018
5	0.522f	0.273	0.252	46.895	0.022	5.265	1	174	0.023
a Measure point = M1 – after the access									
b Predictors: (Constant), treatment model									
c Predictors: (Constant), treatment model, QB									
d Predictors: (Constant), treatment model, QB, artery pressure									
e Predictors: (Constant), treatment model, QB, artery pressure, venous pressure									
f Predictors: (Constant), treatment model, QB, artery pressure, venous pressure, needle									

Table 4B: Model Summary[a]: Measure point = M2 – before the dialyzer									
Model	R	R Square	Adjusted R Square	Std. Error of the Estimate	Change Statistics				
					R Square Change	F Change	df1	df2	Sig. F Change
1	0.486b	0.236	0.232	14.711	0.236	57.991	1	188	<0.001
a Measure point = M2 – before the dialyzer									
b Predictors: (Constant), venous pressure									

Table 4C: Model Summary[a]: Measure point = M3 – after the dialyzer									
Model	R	R Square	Adjusted R Square	Std. Error of the Estimate	Change Statistics				
					R Square Change	F Change	df1	df2	Sig. F Change
1	0.308b	0.095	0.09	2.364	0.095	18.61	1	178	<0.001
2	0.542c	0.293	0.285	2.094	0.199	49.767	1	177	<0.001
3	0.622d	0.387	0.377	1.956	0.094	26.938	1	176	<0.001
4	0.662e	0.439	0.426	1.877	0.051	16.047	1	175	<0.001
5	0.673f	0.453	0.437	1.859	0.014	4.438	1	174	0.037
a Measure point = M3 – after the dialyzer									
b Predictors: (Constant), artery pressure									
c Predictors: (Constant), artery pressure, venous pressure									
d Predictors: (Constant), artery pressure, venous pressure, TMP									
e Predictors: (Constant), artery pressure, venous pressure, TMP, dialysate temperature									
f Predictors: (Constant), artery pressure, venous pressure, TMP, dialysate temperature, type of device									

Table 4D: Model Summary[a]: Measure point = M4 -after the venous chamber									
Model	R	R Square	Adjusted R Square	Std. Error of the Estimate	Change Statistics				
					R Square Change	F Change	df1	df2	Sig. F Change
1	0.383b	0.146	0.142	2.023	0.146	30.514	1	178	<0.001
2	0.410c	0.168	0.158	2.003	0.021	4.543	1	177	0.034
3	0.485d	0.235	0.222	1.926	0.067	15.495	1	176	<0.001
a Measure point = M4 -after venous chamber									
b Predictors: (Constant), venous pressure									
c Predictors: (Constant), venous pressure, artery pressure									
d Predictors: (Constant), venous pressure, artery pressure, type of device									

Supplement Figures:

Supplement Fig 1A

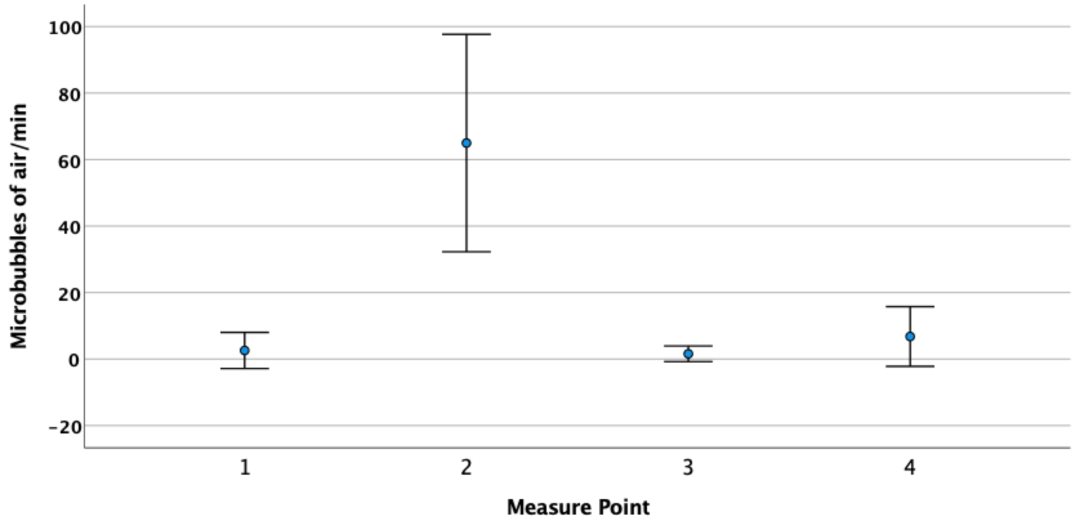

Supplement Figures 1A to C: The figure displays three series of measurements when an Autotest was done while analyzing the median and IQR of microbubbles at site 1 (after access), site 2 (before dialyzer), site 3 (after dialyzer), and site 4 (after the venous air trap). A) with the device AK200 in the postdilution mode. The Autotest was done while measuring site M2.

Supplement Fig 1B

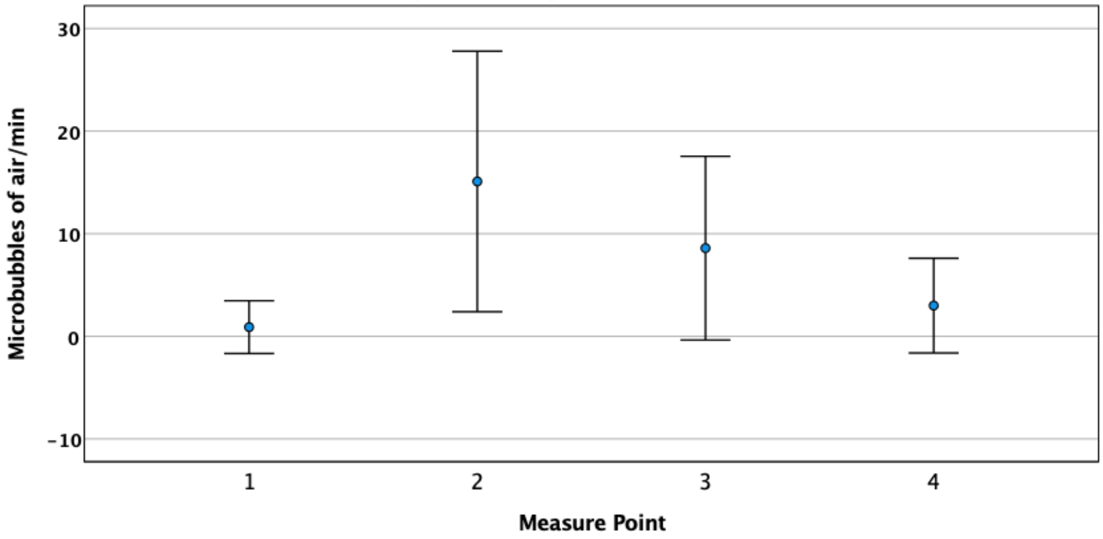

B) with the device Artis in the postdilution mode. The Autotest was done while measuring site M2;

Supplement Fig 1C

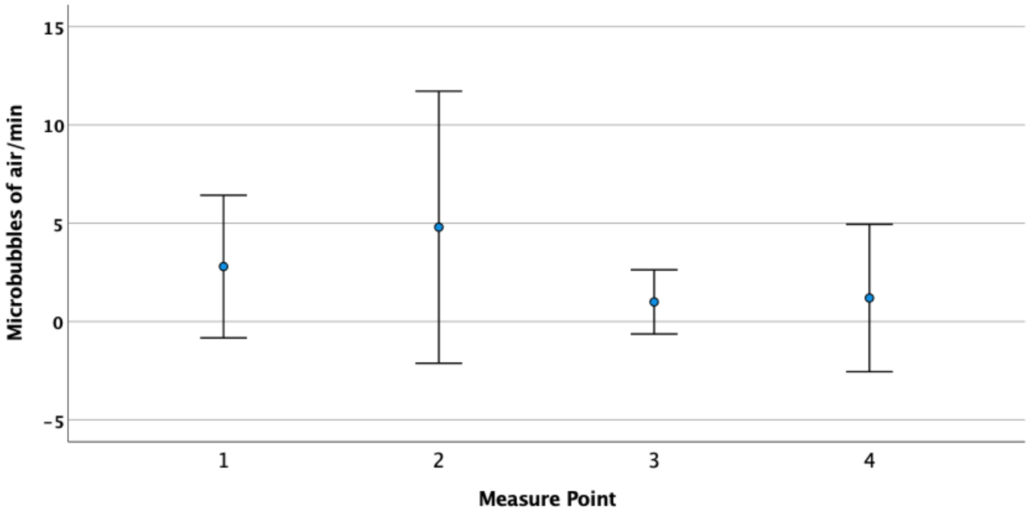

C) with the device Artis in the postdilution mode. The Autotest was done while measuring site M3.

Supplement Fig 2

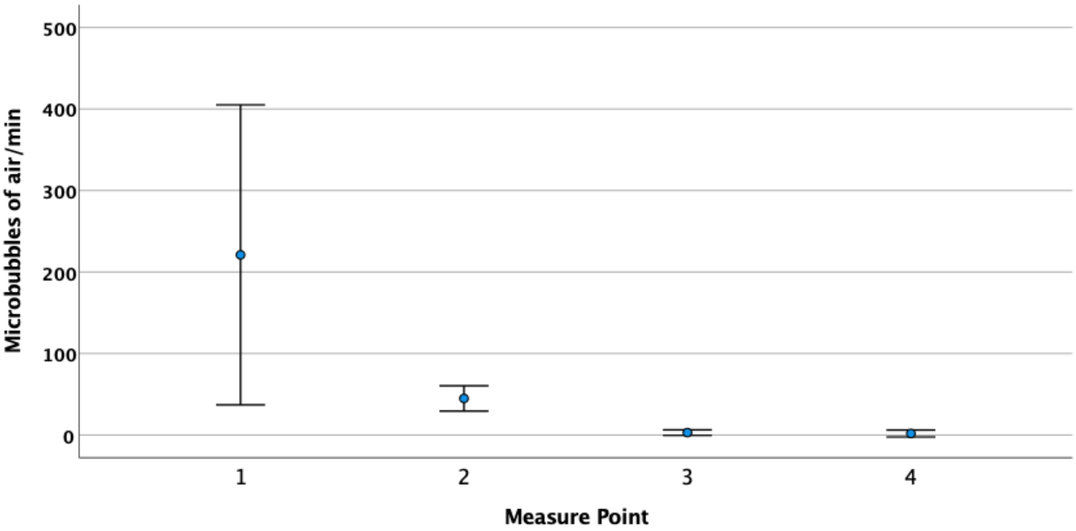

Supplement Figure 2: The figure displays two series of measurement of the median and IQR of microbubbles at site 1 (after access), site 2 (before dialyzer), site 3 (after dialyzer), and site 4 (after the venous air trap). Dialysis with the Artis device in the postdilution mode. High counts of MB at site M1 indicate air leakage at the access site.
